# Practice Trends and Characteristics of US Hospitalists From 2012 to 2018

**DOI:** 10.1001/jamahealthforum.2021.3524

**Published:** 2021-11-05

**Authors:** Kira L. Ryskina, Kaitlyn Shultz, Mark Aaron Unruh, Hye-Young Jung

**Affiliations:** 1 Department of Medicine, Perelman School of Medicine at the University of Pennsylvania, Philadelphia; 2 Leonard Davis Institute of Health Economics, Philadelphia, Pennsylvania; 3 Department of Population Health Sciences, Weill Cornell Medical College, New York, New York

## Abstract

This cohort study uses Medicare data to assess trends and characteristics among hospitalists who shift practice to settings outside of the hospital.

## Introduction

Recent decades have observed a rapid growth in the number of hospitalists.^1,2^ Receiving care from hospitalists has been associated with shorter length of stay and similar outcomes^3^ and lower readmission and mortality rates compared with care from nonhospitalist physicians who were not the patient’s primary care physician.^4^ Furthermore, hospitalist experience (years in practice) has been inversely correlated with patient mortality.^5^ Surveys indicate high turnover among hospitalists compared with other specialties,^6^ but those surveys measure turnover within hospitalist groups rather than the specialty overall. Our objective was to characterize hospitalists who shift practice to settings outside of the hospital.

## Methods

For this cohort study, we used Medicare’s Provider Utilization and Payment Data records from 2012 to 2018, which aggregate Medicare Part B billings by service. This database was linked to physician demographic details and practice characteristics from the Medicare Data on Provider Practice and Specialty files and to county-level characteristics from the Area Health Resource Files. Generalist physicians (internal medicine, geriatrics, general practice, pediatrics, or family medicine) who billed at least 90% of visits from the hospital were considered hospitalists.^4,5^ Physicians with 100 visits or fewer per year were excluded.

Hospitalists were identified in 2012, and those who continued to practice as generalists were followed up through 2018 to examine the settings where they provided care. We measured the proportion of visits in the following settings (service codes in eTable in the Supplement): (1) acute care hospital; (2) office (ie, a location where patients are treated on an ambulatory basis); (3) nursing home or skilled nursing facility; and (4) other (all other settings).

We examined physician characteristics (gender, age, time from medical school graduation, medical school rank), practice characteristics (size of group, Medicare beneficiary volume), and county characteristics (urban location, region, household income, and the proportion of the population who were White). The data sets we used do not contain information on physician race and ethnicity. The characteristics were selected a priori based on availability and relevance in physician workforce literature. We compared the characteristics between physicians who continuously practiced as hospitalists vs those who changed practice in any year by using the χ^2^ test for categorical and the Wilcoxon test for continuous variables. We used multivariable linear probability regression to measure the association between the characteristics and continuous hospitalist practice. We also performed a sensitivity analysis, including in our sample the hospitalists who did not bill Medicare or subspecialized in 2013 to 2018.

Data analyses were conducted from March 15 to June 28, 2021. Two-sided *P* values <.05 were considered significant. Analyses were conducted using SAS, version 9.4 (SAS Institute Inc). The University of Pennsylvania Institutional Review Board waived review of this study and the need for informed consent because the data used were publicly accessible. We followed the Strengthening the Reporting of Observational Studies in Epidemiology (STROBE) reporting guideline for cohort studies.

## Results

Of the 16 985 hospitalists identified in 2012 (mean [SD] age, 41.1 [8.1] years; 6146 women [36.2%] and 10 839 men [63.8%]), 4052 (23.9%) shifted practice to another setting in at least 1 year during the follow-up period. Those physicians tended to be in smaller practices (eg, compared with solo practice, the regression coefficient for practices with ≥300 physicians was 22.06; 95% CI, 18.60-25.52; *P* < .001) and/or rural practices (regression coefficient for urban vs rural, 3.40; 95% CI, 1.28-5.53; *P* = .002) (Table 1). Physicians who changed practice were more likely to split time between a hospital and other settings than to practice predominantly outside of the hospital; eg, after hospitalists, the second largest category (601 of 16 985 [3.5%]) was physicians who provided 50% or more but less than 90% of visits in the hospital, with the other visits provided in other settings (Table 2).

**Table 1.** Baseline Demographic Details and Practice Characteristics of Physicians Who Were Hospitalists in 2012

Characteristic	No. (%)			Regression coefficient (95% CI)^c^	*P* value
	Cohort overall	Always hospitalist^a^	Shifted practice to another setting^b^		
No. (%)	16 985 (100)	12 933 (76.1)	4052 (23.9)	NA	NA
Age, y					
<30	220 (1.3)	170 (1.3)	50 (1.2)	[Reference]	NA
30-39	8224 (48.4)	6371 (49.3)	1853 (45.7)	0.44 (−5.22 to 6.10)	.88
40-49	5904 (34.8)	4487 (34.7)	1417 (35.0)	0.08 (−5.63 to 5.79)	.98
50-59	2168 (12.8)	1589 (12.3)	579 (14.3)	−1.29 (−7.18 to 4.60)	.67
≥60	469 (2.8)	316 (2.4)	153 (3.8)	−4.66 (−11.5 to 2.14)	.18
Gender					
Female	6146 (36.2)	4756 (36.8)	1390 (34.3)	[Reference]	NA
Male	10 839 (63.8)	8177 (63.2)	2662 (65.7)	−0.77 (−2.12 to 0.59)	.27
Top 20 medical school	727 (4.3)	589 (4.6)	138 (3.4)	2.63 (−0.56 to 5.81)	.11
Practice size, No. of physicians^d^					
Solo practice	746 (4.4)	445 (3.4)	301 (7.4)	[Reference]	NA
2-9	1288 (7.6)	804 (6.2)	484 (11.9)	2.46 (−1.43 to 6.34)	.22
10-49	4550 (26.8)	3366 (26.0)	1184 (29.2)	13.24 (9.87 to 16.60)	<.001
50-99	2382 (14.0)	1842 (14.2)	540 (13.3)	16.50 (12.92 to 20.08)	<.001
100-299	4399 (25.9)	3466 (26.8)	933 (23.0)	18.01 (14.62 to 21.40)	<.001
≥300	3620 (21.3)	3010 (23.3)	610 (15.1)	22.06 (18.60 to 25.52)	<.001
Annual Medicare beneficiary volume, mean (SD)^e^	379.0 (212.5)	379.4 (208.4)	377.5 (225.3)	0.06 (0.03 to 0.09)	<.001
Region^f^					
Northeast	3684 (21.7)	2870 (22.2)	814 (20.1)	[Reference]	NA
Midwest	3777 (22.2)	2898 (22.4)	879 (21.7)	−0.59 (−2.56 to 1.38)	.56
South	6291 (37.0)	4720 (36.5)	1571 (38.8)	−2.01 (−3.87 to 0.14)	.03
West	3193 (18.8)	2428 (18.8)	765 (18.9)	−0.78 (−2.82 to 1.27)	.46
Urban (vs rural)	15 112 (89.0)	11 626 (89.9)	3486 (86.0)	3.40 (1.28 to 5.53)	.002
Median household income, mean (SD), $	53 216 (13 210)	53 326 (13 161)	52 865 (13 364)	−0.002 (−0.05 to 0.05)	.94
Proportion of county population who are White, mean (SD)	71.1 (15.8)	70.8 (15.9)	72.0 (15.6)	−0.10 (−0.14 to −0.05)	<.001

Abbreviations: MD-PPAS, Medicare Data on Provider Practice and Specialty; NA, not applicable.

^a^ An “always hospitalist” is defined as a physician in the cohort who continuously practiced as a hospitalist (billing ≥90% of patient visits from an acute care hospital).

^b^ Represents physicians in the cohort who were hospitalists in 2012 and shifted practice to another setting or settings in any of the study years (billing <90% of patient visits from an acute care hospital).

^c^ Multivariable linear probability models were used to measure the probability of a physician remaining in hospitalist practice throughout the study period.

^d^ Physician group size was measured by summing the number of National Provider Identifiers associated with each Tax Identification Number using the MD-PPAS.

^e^ Patient volume was measured using the number of unique Medicare beneficiaries.

^f^ Physicians practicing in US territories were not categorized into regions (n = 40).

**Table 2.** Practice Settings of Physicians Who Were Hospitalists in 2012 by Year of Follow-up

Setting	Description	No. (%) of physicians (N = 16 985)					
		2013	2014	2015	2016	2017	2018
Hospitalist	≥90% of visits to hospitalized patients	15 997 (94.2)	15 378 (90.5)	14 987 (88.3)	14 603 (86.0)	14 396 (84.8)	14 164 (83.4)
Hospital-SNF practice	≥50% to <90% of visits to hospitalized patients, followed by visits to SNF as the second largest visit type	255 (1.5)	360 (2.1)	424 (2.5)	504 (3.0)	473 (2.8)	454 (2.7)
Hospital-office practice	≥50% to <90% of visits to hospitalized patients, followed by office visits as the second largest visit type	165 (1.0)	213 (1.3)	223 (1.3)	272 (1.6)	263 (1.6)	271 (1.6)
Hospital-other practice	≥50% to <90% of visits to hospitalized patients, followed by “other”^a^ visits as the second largest visit type	335 (2.0)	438 (2.6)	445 (2.6)	492 (2.9)	519 (3.1)	601 (3.5)
SNF practice	≥50% of visits to SNF patients	34 (0.2)	77 (0.5)	129 (0.8)	153 (0.9)	203 (1.2)	254 (1.5)
Office practice	≥50% of visits provided in outpatient office or clinic	53 (0.3)	155 (0.9)	224 (1.3)	335 (2.0)	388 (2.3)	415 (2.4)
Other practice	≥50%of visits provided in “other” settings	73 (0.4)	188 (1.1)	275 (1.6)	333 (2.0)	423 (2.5)	462 (2.7)
Mixed	Physician provided services in ≥3 settings with <50% of services provided in each setting	73 (0.4)	176 (1.0)	278 (1.6)	293 (1.7)	320 (1.9)	364 (2.1)

Abbreviation: SNF, skilled nursing facility.

^a^ Includes all other settings not otherwise specified.

Our sensitivity analyses that included physicians who did not bill Medicare (n = 2994) or subspecialized (n = 2644) in any year during study follow-up were generally consistent with the primary analyses. In addition to smaller practice size and rural location, physicians who shifted practice were more likely to be aged 60 years or older (7.1% vs 2.4%, *P* < .001) and less likely to come from a top 20 medical school (3.6% vs 4.6%, *P* < .001) compared with physicians who continuously practiced as hospitalists, which were not statistically significant coefficients in our primary analysis.

## Discussion

This study found that between 2012 and 2018, nearly 1 in 4 hospitalists shifted practice to other settings, although most hospitalists continued to practice in the hospital at least part time. Our findings have important implications for hospitals, especially during infectious disease outbreaks, such as the COVID-19 pandemic, that place extra demands on hospital-based clinicians. Understanding the drivers behind these trends may help mitigate the high costs of physician turnover in health systems.

Limitations of this study include the use of Medicare fee-for-service claims but not claims for other payers. However, Medicare is the most common payer for inpatient care in the US.

## Notes

Supplement. **eTable.** HCPCS Codes Used to Identify Practice Settings
