# Supplementary material for: Practice Trends and Characteristics of US Hospitalists From 2012 to 2018
Source: JAMA Health Forum. 2021 Nov 5;2(11):e213524. doi: 10.1001/jamahealthforum.2021.3524 (PMC8796912; doi:10.1001/jamahealthforum.2021.3524)
Supplement: Supplement. — eTable. HCPCS Codes Used to Identify Practice Settings [file jamahealthforum-e213524-s001.pdf]

Supplemental Online Content

Ryskina KL, Shultz K, Unruh MA, Jung HY. Practice trends and characteristics of US hospitalists from 2012 to 2018. *JAMA Health Forum.* 2021;2(11):e213524. doi:10.1001/jamahealthforum.2021.3524

eTable. HCPCS Codes Used to Identify Practice Settings

This supplemental material has been provided by the authors to give readers additional information about their work.

© 2021 Ryskina KL et al. *JAMA Health Forum.*

eTable. HCPCS[a] Codes Used to Identify Practice Settings

Setting	HCPCS Codes
Hospital	"99221", "99222", "99223", "99224", "99225", "99226", "99231", "99232", "99233", "99238", "99239", "99217", "99218", "99219", "99220", "99234", "99235", "99236", "99291", "99292", "94003", "94002", "94660", "94662"
Office	“99201", "99202", "99203", "99204", "99205", "99211", "99212", "99213" "99214", "99215"
Nursing home or skilled nursing facility	"99304", "99305", "99306", "99307", "99308", "99309", "99310", "99315", "99316", "99318"
Other	All other HCPCS E&M[b] codes

[a] HCPCS stands for Health Common Procedure Coding System service code.

[b] E&M codes refer to evaluation and management services typically provided by physicians.

© 2021 Ryskina KL et al. *JAMA Health Forum.*
